# Splice-disrupt genomic variants in prostate cancer

**DOI:** 10.1007/s11033-022-07257-9

**Published:** 2022-03-14

**Authors:** Ibrahim O. Alanazi, Salman F. Alamery, Esmaeil Ebrahimie, Manijeh Mohammadi-Dehcheshmeh

**Affiliations:** 1 National Center for Biotechnology, Life Science and Environment Research Institute, King Abdulaziz City for Science and Technology (KACST), Riyadh, Saudi Arabia; 2 Department of Biochemistry, College of Science, King Saud University, Riyadh, Saudi Arabia; 3 Genomics Research Platform, School of Life Sciences, La Trobe University, Melbourne, VIC 3086 Australia; 4 School of Animal and Veterinary Sciences, The University of Adelaide, Adelaide, 5371 Australia; 5 School of BioSciences, The University of Melbourne, Melbourne, VIC 3010 Australia

**Keywords:** Genomic mutation, Prostate cancer, Systems biology, Splicing, Variant discovery

## Abstract

**Background:**

Splice-disrupt genomic variants are one of the causes of cancer-causing errors in gene expression. Little is known about splice-disrupt genomic variants.

**Methods and results:**

Here, pattern of splice-disrupt variants was investigated using 21,842,764 genomic variants in different types of prostate cancer. A particular attention was paid to genomic locations of splice-disrupt variants on target genes. *HLA-A* in prostate cancer, *MSR1* in familial prostate cancer, and *EGFR* in both castration-resistant prostate cancer and metastatic castration-resistant had the highest allele frequencies of splice-disrupt variations. Some splice-disrupt variants, located on coding sequences of *NCOR2*, *PTPRC*, and *CRP*, were solely present in the advanced metastatic castration-resistant prostate cancer. High-risk splice-disrupt variants were identified based on computationally calculated Polymorphism Phenotyping (PolyPhen), Sorting Intolerant From Tolerant (SIFT), and Genomic Evolutionary Rate Profiling (GERP) + + scores as well as the recorded clinical significance in dbSNP database of NCBI. Functional annotation of damaging splice-disrupt variants highlighted important cancer-associated functions, including endocrine resistance, lipid metabolic process, steroid metabolic process, regulation of mitotic cell cycle, and regulation of metabolic process. This is the first study that profiles the splice-disrupt genomic variants and their target genes in prostate cancer. Literature mining based variant analysis highlighted the importance of rs1800716 variant, located on the *CYP2D6* gene, involved in a range of important functions, such as RNA spicing, drug interaction, death, and urotoxicity.

**Conclusions:**

This is the first study that profiles the splice-disrupt genomic variants and their target genes in different types of prostate cancer. Unravelling alternative splicing opens a new avenue towards the establishment of new diagnostic and prognostic markers for prostate cancer progression and metastasis.

**Supplementary Information:**

The online version contains supplementary material available at 10.1007/s11033-022-07257-9.

## Introduction

Alternative RNA splicing is an emerging topic in molecular and clinical oncology [1, 2]. Alternative splicing is the key mechanism to generate many mRNA transcripts from the relatively low number of human genes, which can lead to the assembly of different protein isoforms with distinct functions. This structural modification of gene transcripts and their encoded proteins is considered a vital process that increases diversity of protein functions to generate the complex cellular proteome [3, 4]. The outcome of alternative splicing can result in a complete loss of function or the acquisition of new functions [3, 4]. In humans, it is estimated that up to 94% of genes undergo alternative splicing, resulting in more than 100,000 transcripts [5–7]. Accumulating evidence highlights the importance of study of gene and protein in parallel with alternative splice variants [8].

Dysregulation of post-transcriptional regulation can result in defective proteins or transcripts without causing genetic diseases [9]. Alternative pre-mRNA splicing leads to distinct products of gene expression in normal development and disease. Here, we observed higher variation in GERP++ score splice-disrupt variants of progressive prostate cancer compared to the non-progressive one. Antagonistic splice variants of genes involved in differentiation, apoptosis, invasion and metastasis often exist in a delicate equilibrium that is found to be perturbed in tumors.

Precise pre-mRNA splicing is vital for correct protein translation. Precise pre-mRNA splicing is related to the presence of consensus “cis” sequences, identifying exon-intron boundaries and regulatory sequences rby splicing machinery [10]. Point mutations may occur in both introns and exons disrupting existing splice sites or splicing regulatory sequences, generating new transcripts, even pathogenic ones [10]. Splice-disrupt genomic mutations can also be a source of cancer-causing errors in gene expression resulting in cancer-specific alternative splicing [11]. It has been demonstrated that nearly half of all active alternative splicing events are altered in ovarian and breast tumour cells compared to normal tissue [4]. Cancer can occur irrespective of changes in expression of a gene or protein, but rather because of aberrant splice variants that are linked to cancer progression and/or drug resistance and is compensated by the decreased expression of other splice variants originating from that same gene.

Within different types of cancer-associated genomic variants, splice-disrupt genomic variants are the less studied, particularly in prostate cancer. There is an ample evidence of the discovered point mutations that prevent appropriate splicing by disruption of exonic splicing enhancers [12]. Point mutation in the splicing acceptor or donor site can lead to the production of an altered mature mRNA and can result in intron retention, exon skipping, or alternative 3′ and 5′ splicing site [13]. Even, missense mutations or silent substitutions that do not alter protein function can influence on pre-mRNA splicing and have undiscovered pathological functions [12, 14]. In multiple sclerosis, splice-disrupt variants have been identified on 27 immune-related and myelin genes [15]. Splice-disrupt variants on *NBAS, SLC16A1*, *RHD*, *PNPLA2* have been accounted as genetic basis of recurrent liver failure, ketoacidosis, variant D phenotype, progressive severe myopathy, respectively [16–19]. Pathogenic consequences of splice-disrupt variants in *CHEK2* genes in many cancers has been documented in individuals carrying a single pathogenic splice-disrupt variant [13].

Our knowledge about abnormal splice-disrupt genomic variants in prostate cancer, and their prospective contribution to cancer progression (castration-resistant prostate cancer and metastatic castration-resistant prostate cancer) is limited. Furthermore, their relative abundance and their potential to use splice-disrupt in cancer diagnosis and treatment has been largely neglected [20].

Whole genome sequencing projects, particularly 1000 Genomes Project, have resulted in bulk identification of human variations, including splice-disrupt variants [21]. The identified variants progressively deposit in major public repositories of genomic variants, noticeably dbSNP, the NCBI database of genetic variation, to be employed by the researchers and clinicians worldwide [22]. The deposited data is a great resource to shed light on the possible involvement of splicing events in human diseases [2, 20].

For the first time, we mined a large dataset of genomic variants in different types of prostate cancer, identifing the splice-disrupt variants and their target genes. Furthermore, we investigated the association between splice-disrupt variants and advanced types of prostate cancer. Finally, computational systems biology was applied to investigate the functional consequences of the discovered splice-disrupt variants.

## Methods

### Identification of splice-disrupt variants in different types of prostate cancer and their genomic locations

The National Center for Biotechnology Information (NCBI) database of genetic variation (dbSNP database) [22] was used as the main resource for gathering of variants. Variants were retrieved for common and advanced types of prostate cancer (biological associations), including prostate cancer (PC), castration-resistant prostate cancer (CRPC), familial prostate cancer (FPC), and metastatic castration-resistant prostate cancer (MCRPC). SQL-based Pathway Studio Web tool (Elsevier) was used for navigating and downloading variants from dbSNP, as previously described [20].

Translational impact of variants including missense, splice-disrupt, coding sequences insertion or deletion (CDs indel), nonsense, misstart, and non-stop were retrieved and variants were filtered for splice-disrupt variants using SQL Table of Pathway Studio tool. As a result, splice-disrupt variants were identified for different types of prostate cancer (PC, CRPC, FPC, and MCRPC).

### Characterising of splice-disrupt variants

#### Variant (allele) frequency

Allele (variant) frequency stands for a gene variant (an allele) in a specific locus in a population, commonly presented as percentage/fraction. Minor allele frequency explains the frequency where the second most common allele occurs in a population; for example, allele frequency of 1% mean that the variant happens in 1% of population. We used 1000 Genomes Project [21] to extract the minor allele frequency using Pathway Studio tool. No cutoff was used for minor allele frequency of splice-disrupt variants in this study. This allowed us to investigate the contribution of less frequent (1–5% minor allele frequency) and rare variants (< 1% minor allele frequency) to different types of prostate cancer.

#### Genomic locations

Genomic locations of variants were identified based on dbSNP, and variants were assigned to the following locations: CDs (coding sequences), 3′UTR (untranslated region), 5′UTR, intergenic, and intronic variants.

### Scoring of splice-disrupt variants and identifying of high-risk splice-disrupt variants

High-risk splice-disrupt variants were identified using dbNSFP [23–25], a database of human non-synonymous SNVs and their functional predictions as well as dbSNP. Scoring of splice-disrupt variants was performed using computationally calculated Polymorphism Phenotyping (PolyPhen), Sorting Intolerant From Tolerant (SIFT), and Genomic Evolutionary Rate Profiling (GERP)++ scores from dbNSFP as well as the recorded clinical significance in dbSNP database of NCBI.

#### SIFT score

SIFT algorithm is developed to predict the effect of coding variants on protein function based on sequence homology and the physical properties of amino acids. SIFT is considered as a standard tool for characterizing missense variation [26, 27]. SIFT score is an important computational functional measurement predicting whether an amino acid substitution is deleterious [28]. SIFT values were calculated for splice-disrupt variants located on CDs. We categorized the variants based on the cutoff of 0.05 as: (1) Tolerable (SIFT score > 0.05) and (2) Damaging (SIFT score ≤ 0.05).

#### PolyPhen score

PolyPhen algorithm, like SIFT, use sequence homology of related proteins to evaluate whether an amino acid substitution can be deleterious to protein function based on the degree of conservation of the affected base throughout evolution. SIFT relies solely on sequence homology [29] while PolyPhen employs annotated UniProt entries to evaluate whether the amino acid substitution happens within an important structural or functional site of the protein, such as active or binding sites, and residues involved in disulphide bond formation [30, 31]. PolyPhen scores were classified in 3 levels as: (1) Benign (PolyPhen score ≤ 0.452), (2) Possibly Damaging (0.452 < PolyPhen score < 0.957), and Probably Damaging (PolyPhen score ≥ 0.957).

#### GERP++ conservation score

GERP is an evolutionary conservation score which have a good correspondence with clinical significance and pathogenicity level. GERP++ demonstrates the constrained elements in multiple alignments by quantifying substitution deficits. These deficits identify substitutions that would have happened if the element were neutral DNA, but did not happen as the element has been experienced functional constraint [32]. Low values of GERP++ score stand for low level of conservation and high values for high level of conservation.

#### Clinical significance

Pathogenic status of splice-disrupt variants was obtained from dbSNP database. dbSNP is the main global reference and repository of single nucleotide variations [22, 33, 34]. Assertions of clinical significance for alleles of human sequence variations are provided by the submitter at the time of submission of variant to NCBI, as: non-pathogenic, probable pathogenic, pathogenic, drug response, histocompatibility, untested, and unknown. Pathogenetic status is also supported by associated publications (citations) in dbSNP.

### Functional annotation of splice-disrupt variants

Genes targeted by splice-disrupt variants in each type of prostate cancer (PC, CRPC, FPC, and MCRPC) were used as input for Gene ontology (GO) enrichment analysis in three terms of biological process (BP), molecular function (MF), and cellular component (CC). To this end, the following tools were employed: (1) Comparative GO [35, 36], and (2) STRING [37].

### Literature-mining network of splice-disrupt variants, their annotated genes, and interactions with different types of prostate cancer

Literature mining more was used as a validation of in silico discovered splice-disrupt variants, to to investigate which of the computationally selected splice-disrupt variants have evidence of involvement in different types of prostate cancer.

Literature mining was performed using Pathway Studio Mammal database (Elsevier) [38], Version 12.4.0.3, as recently described [39]. Database contains functional relationships and pathways of mammalian proteins, covering human, mice, and rat. The database is enriched with protein-drug interaction and protein-disease interaction databases, called ChemEffect and DiseaseFx [40], respectively. The database is compiled using Medscan technology [41], a natural language processing engine, by text mining of over 24,000,000 PubMed abstracts and over 3,500,000 Elsevier and 3rd party full-text papers. The database is enriched with variation databases dbSNP v145 and dbNSFP v2.9, providing the opportunity to discover and visualise the relationships between genomic variants, genes, clinical parameters, diseases, and chemicals (small molecules).

### Statistical analysis

Analysis of Variance (ANOVA) followed by Mean comparison using Tukey test was performed to compare the allele frequency of GERP++ conservation score between different types of prostate cancer. Two-sample proportion test was used to compare the clinical significance (occurrence of pathogenic status) between types of prostate cancer. Leven’s test was used to compare variance of allele frequency, and GERP++ between types of prostate cancer. Analysis was performed in MINITAB 18 (https://www.minitab.com). Graphs were visualized using GraphPad Prisim 7 (https://www.graphpad.com/).

## Results

### Mined splice-disrupt variants in prostate cancer (PC), castration-resistant prostate cancer (CRPC), familial prostate cancer (FPC), and metastatic castration-resistant prostate cancer (MCRPC)

Variant analysis resulted in identification of 854, 24, 112, and 35 splice-disrupt variants in PC, FPC, CRPC, and MCRPC. As presented in Supplementary 1, *HLA-A* in PC, *MSR1* in FPC, and *EGFR* in both CRPC and MCRPC had the highest allele frequencies of splice-disrupt variations. Supplementary 1 presents list of variants in different types of prostate cancer based on whole genome sequencing.

### Genomic locations of splice-disrupt variants

**Fig. 1:**
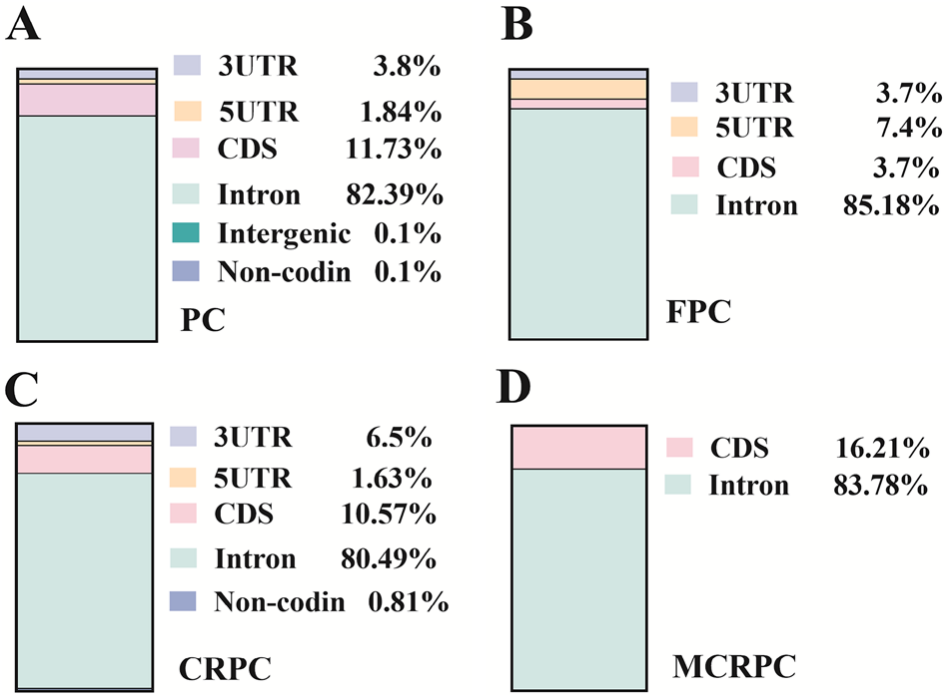
Genomic distribution of splice-disrupt variants in different types of prostate cancer. **A**—PC (prostate cancer), **B**—FPC (familial prostate cancer), **C**—CRPC (castration-resistant prostate cancer), and **D**—MCRPC (metastatic castration-resistant prostate cancer)

Genomic locations of splice-disrupt variants are presented at Fig. 1. As it can be inferred from Fig. 1, there is a remarkable difference in genomic locations of splice-disrupt variants in different types of prostate cancer. Splice-disrupt variants in MCRPC are only located on CDs and Intron. MCRPC has the highest percentage of CDs variants (16.21%) compared to the other types of prostate cancer. FPC has remarkably high enrichment of 5′UTR variants (7.4%). CRPC has the highest occurrence of 3′UTR splice-disrupt variants (6.5%) demonstrating the possible involvement of variants affecting the expression level and possible involvement of 3′UTR.

### Identifying the high-risk splice-disrupt variants based on PolyPhen, SIFT, and GERP++ scores as well as clinical significance and their associated mechanisms

**Fig. 2:**
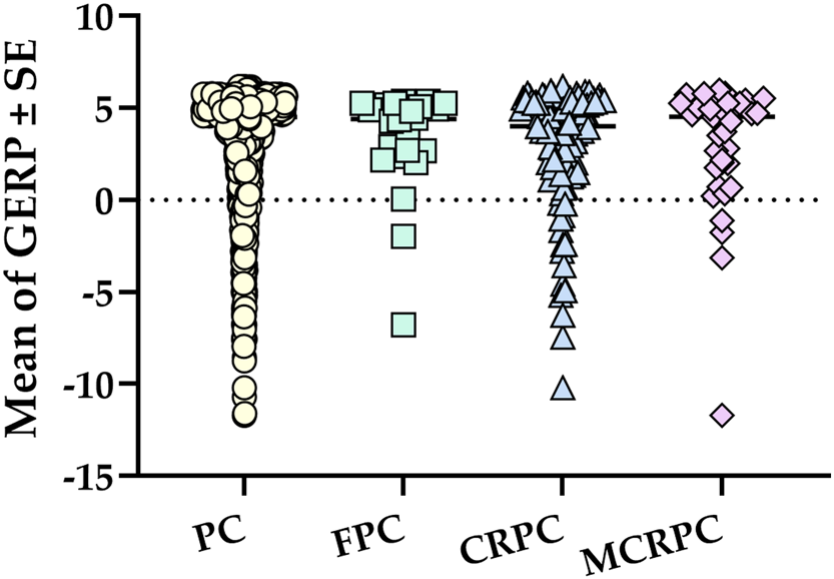
Comparison of GERP score of splice-disrupt genomic variants between different types of prostate cancer (PC: prostate cancer, FPC: familial prostate cancer, CRPC: castration-resistant prostate cancer, and MCRPC: metastatic castration-resistant prostate cancer

High-risk splice-disrupt variants were selected based on low SIFT score, high PolyPhen score and high GERP++ score as well as reported pathogenic clinical significance (retrieved from dbSNP database) (Supplementary 2–5). In more advanced type of prostate cancer (MCRPC), splice-disrupt variants, located on CDs of *NCOR2*, *PTPRC*, and *CRP*, are the high-risk variants. In CRPC, variants located on CDs of *INSRR*, *MAEA*, *ESR1*, *TACC2*, *RB1*, *CRP* as well as Intron based variants on *BRCA1* are the high-risk ones. *BRCA1*, *MLH1*, *MSR1*, *CYP1A1*, *CHEK2*, and *ELAC2* received the highest GERP++ score in FPC. *INSRR*, *MDC1*, *WWOX*, *MAEA*, *FKBP5*, *TNFRSF10C, FAM13C*, *ESR1*, *CYP27A1*, and *BRCA1* are the top variants in PC. Figure 2 compares the overall GERP score in different types of PC where the highest score belongs to FPC documenting that this type of cancer has more simple genetic background, less affected by environmental conditions.

**Fig. 3:**
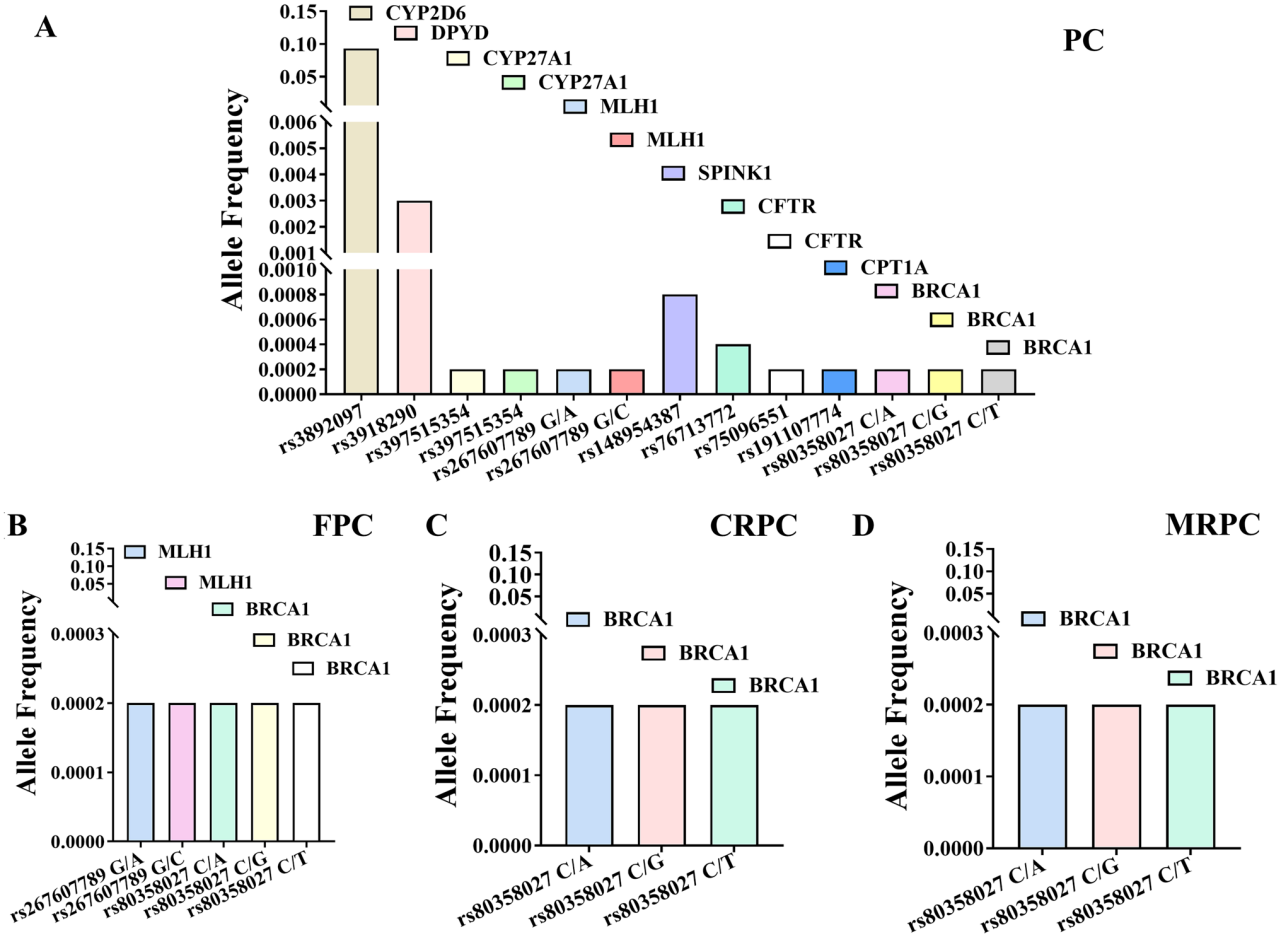
Highly pathogenic splice-disrupt genomic variants and their corresponding genes in different types of prostate cancer. **A**—PC (prostate cancer), **B**—FPC (familial prostate cancer), **C**—CRPC (castration-resistant prostate cancer), and **D**—MCRPC (metastatic castration-resistant prostate cancer)

Highly pathogenic splice-disrupt genomic variants and their corresponding genes in different types of prostate cancer (PC, CRPC, FPC, and MCRPC are presented in Fig. 3. Clinical significance of human sequence variations was obtained from dbSNP. *BRCA1* in all types of prostate cancer was the target of pathogenic splice-disrupt variants. Interestingly, *CYP2D6* genes harbors splice-disrupt variants with high allele frequency.

### Intersection of splice-disrupt variants in different types of prostate cancer

**Fig. 4:**
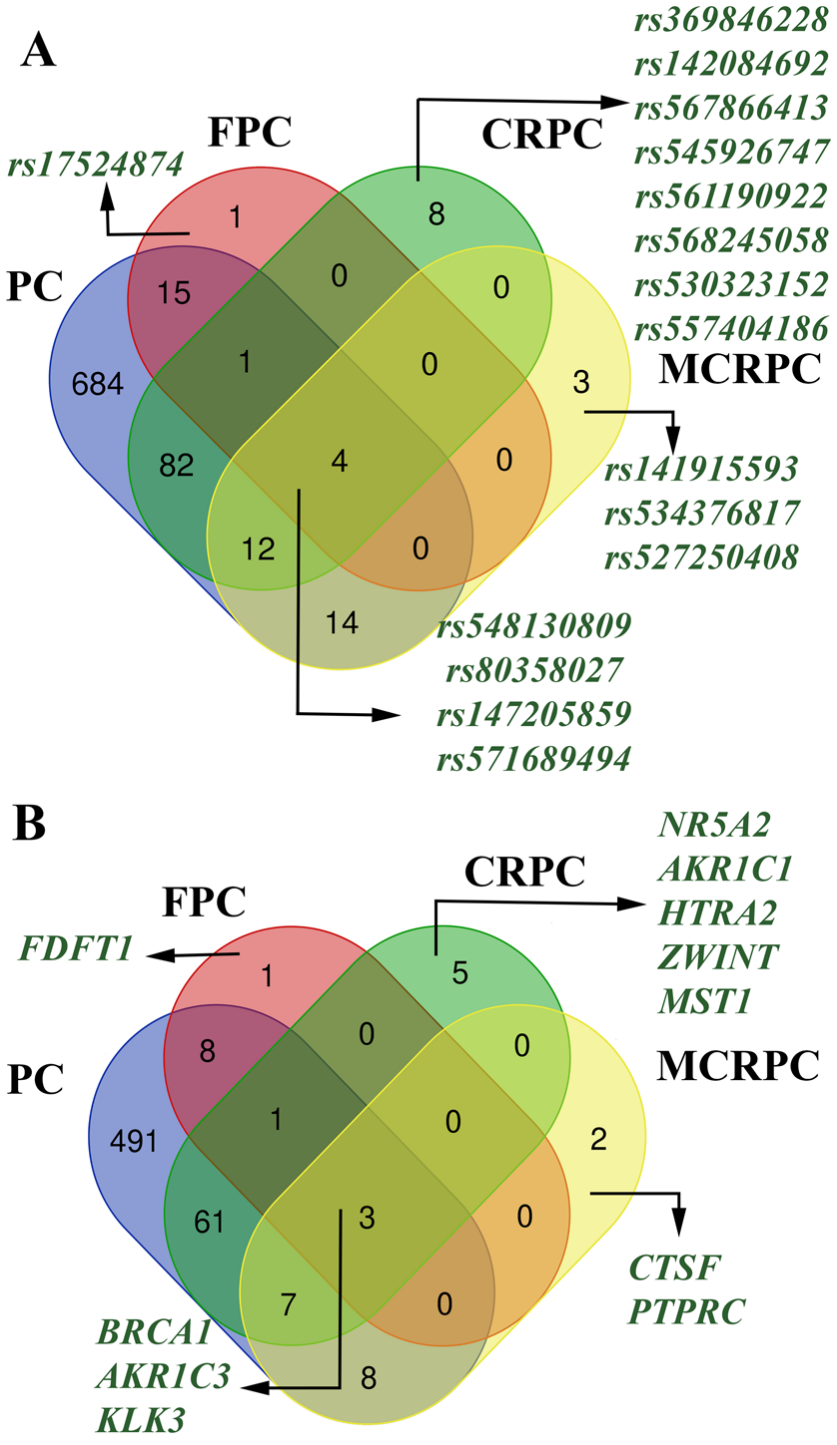
Overlapping splice-disrupt genomic variants and their target genes between different types of prostate cancer. **A** Splice-disrupt variants that are shared between different types of prostate cancer or are unique to a particular type of prostate cancer (specific splice-disrupt variants). **B** Genes that harbor splice-disrupt genomic variants in different types of prostate cancer. *PC* prostate cancer, *CRPC* castration-resistant prostate cancer, *FPC* familial prostate cancer, *MCRPC* metastatic castration-resistant prostate cancer. Total number of splice-disrupt genomic variants was 884 in PC, 24 in FPC, 112 in CRPC, and 35 in MCRPC

Common and specific splice-disrupt genomic variants between different types of prostate cancer (PC, CRPC, FPC, and MCRPC) is presented in Fig. 4. Splice-disrupt variants on *BRCA1*, *AKR1C3*, and *KLK3* is observed in all types of prostate cancer. Splice-disrupt variants on *CTSF* and *PTPRC* is specific to MCRPC and may contribute to prostate cancer progression. *FDFT1* solely happens in FPC. Splice-disrupt variants on *NR5A2*, *HTRA2*, *AKR1C*, *ZWINT*, and *MST1* are linked to CRPC.

### Progressive type of prostate cancer has higher diversity (variance) of GERP++ score in CDs

**Fig. 5:**
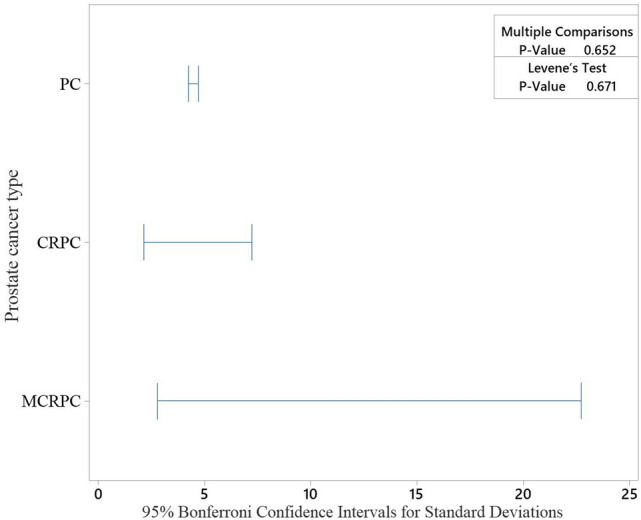
Comparison of variation in GERP++ score of CDs (coding sequences)-located splice-disrupt variants in common and advanced types of prostate cancer. PC, CRPC, and MCRPC. Leven’s test was used for statistical comparison. FPC had only one splice-disrupt variants on CD. Consequently, it was not possible to calculate variance for FPC

Variance analysis of CDs-located splice-disrupt variants showed that advanced type of prostate cancer, MCRPC, has remarkable higher variation in GERP++ score (Fig. 5). High diversity of GERP++ score in MCRPC demonstrates the more complex nature and diverse genomic background of MCRPC and highlights the importance of splice-disrupt variants in progressive prostate cancer.

### Functional annotation of damaging splice-disrupt variants

Supplementary 6 presents functional annotation of high-risk splice-disrupt variants in different types of prostate cancer by enrichment analysis using GO database as well as KEGG pathways. According to KEGG pathway analysis, damaging splice-disrupt variants are significantly (p < 0.01) involved in Endocrine resistance and different types of cancer.

Lipid metabolic process, steroid metabolic process, regulation of mitotic cell cycle, negative regulation of metabolic process, negative regulation of signal transduction, and response to lipid are the key Biological Processes enriched by damaging splice-disrupt variants. One of the significant functions were negative regulation of production of miRNAs involved in gene silencing which variants on *ESR1* are *NCOR1* are involved in this function that can explain the link between UTR damaging variants and microRNA regulation.

### Literature-mining based identification of splice-disrupt variants, their annotated genes, and interactions with different types of prostate cancer

**Fig. 6:**
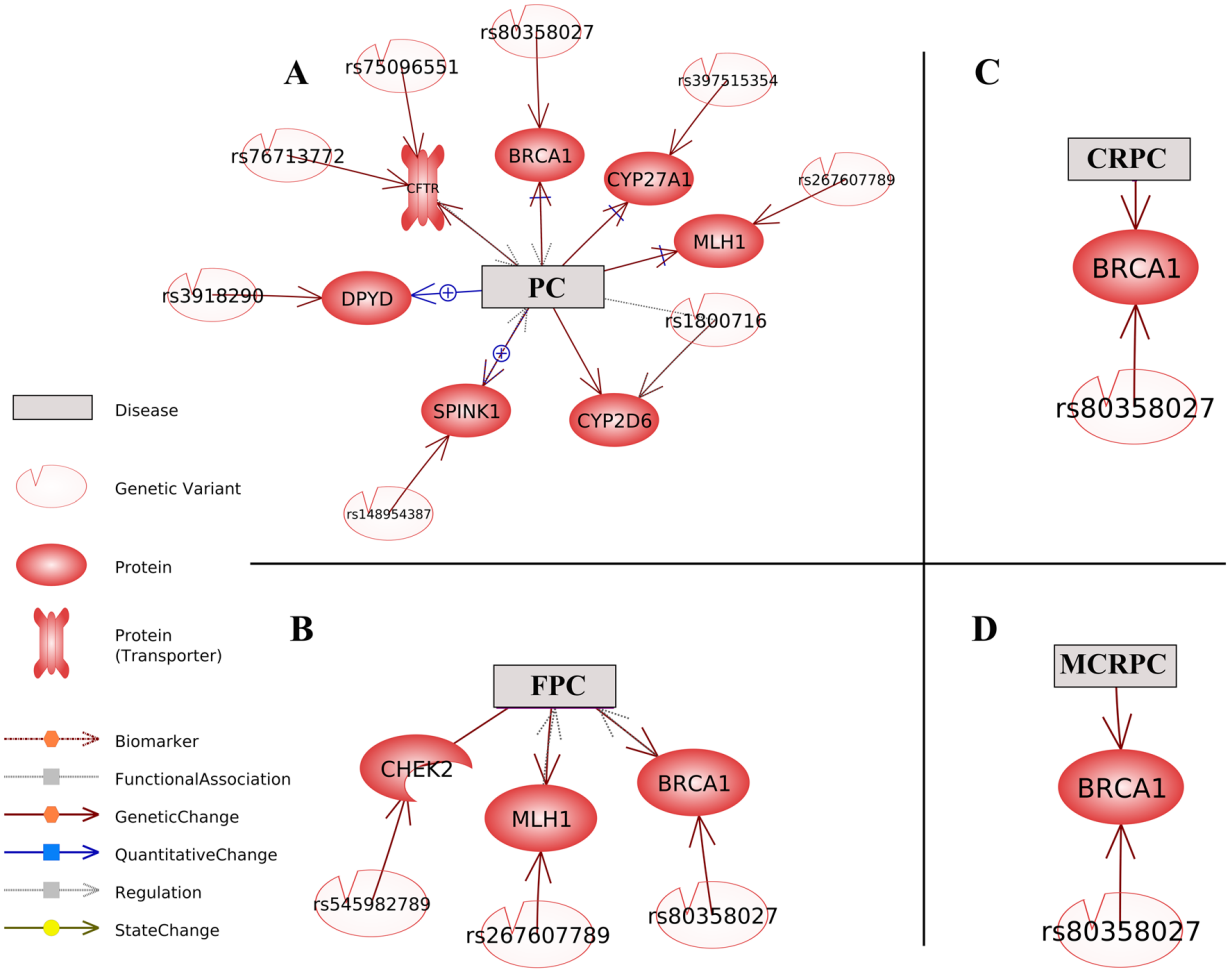
Literature-mining based identification of splice-disrupt genomic variants, their annotated genes, and their interactions with different types of prostate cancer. **A** Literature mining based network of splice-disrupt variants and their target genes in PC. **B** Literature mining based network of splice-disrupt variants and their target genes in FPC. **C** Literature mining based network of splice-disrupt variants and their target genes in CRPC. **D** Literature mining based network of splice-disrupt variants and their target genes in MCRPC. Mined references underpinning the Literature-mining network is presented in Supplementary 7

**Fig. 7:**
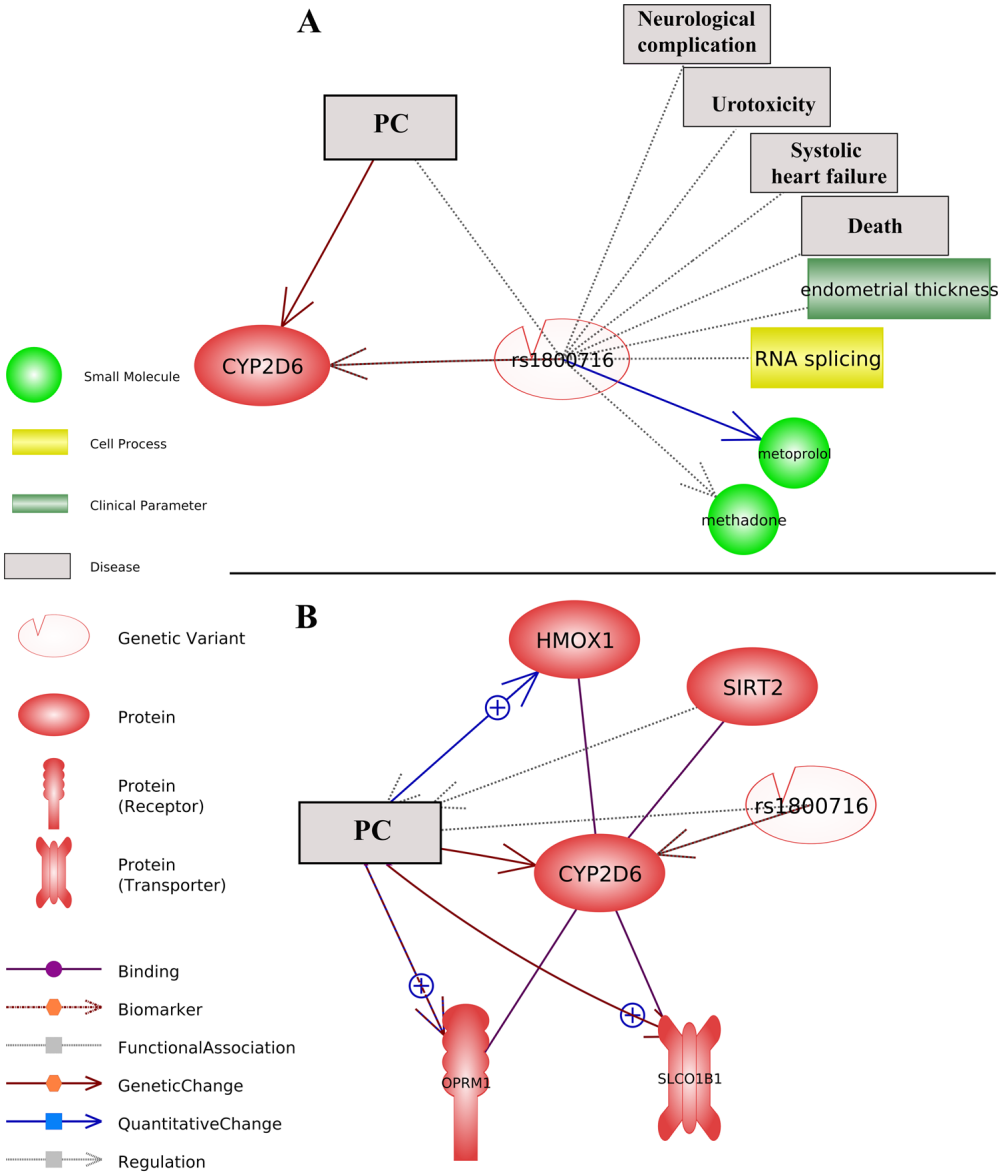
rs1800716 splice-disrupt variant, located on the *CYP2D6*, is involved in a range of important functions, such as RNA spicing, drug interaction, death, and urotoxicity. **A** Interaction of rs1800716 with clinical parameters, disease, small molecules (chemical/drugs). **B** Gene interaction network of *CYP2D6*, the target gene of rs1800716 variant with the other genes. Mined references underpinning the literature-mining network of rs1800716 is presented in Supplementary 8

Literature-mining based identification of variants, their annotated genes, and interactions with different types of prostate cancer is presented in Fig. 6. Mined references underpinning the network is presented in Supplementary 7. BRCA1 splice-disrupt variants are involved in different types of prostate cancer (Fig. 6). rs545982789 splice-disrupt on *CHEK2* kinase is an important genetic change involved in FPC (Fig. 7). Literature mining based variant analysis highlighted the importance of rs1800716 variant, located on the *CYP2D6* gene, involved in a range of important functions, such as RNA spicing, drug interaction, death, and urotoxicity.

## Discussion

Alternative splicing is one of the complexities of systems biology, particularly in cancer studies. Compared to the other type of splice variants, little is known about splice-disrupt genomic variants in cancer. Here, we developed a bioinformatic pipeline for extraction and detection of high-risk splice-disrupt genomic variants, by analysis of big data of deposited variants in prostate cancer, castration-resistant prostate cancer, familial prostate cancer, and metastatic castration-resistant prostate cancer. We showed that some splice-disrupt variants are solely present in the advanced metastatic castration-resistant prostate cancer. This is the first study that profiles the splice-disrupt genomic variants and their target genes in different types of prostate cancer. In final step, literature mining was used to uncover and visualise the relationships between splice-disrupt variants, genes, clinical parameters, diseases, and chemicals (small molecules), highlighting the importance of rs1800716 splice-disrupt variants, located on *CYP2D6*, in prostate cancer.

Noticeably, no cutoff was used for minor allele frequency of splice-disrupt variants in this study. This allowed us to investigate the contribution of less frequent (1–5%, minor allele frequency) and rare variants (< 1% minor allele frequency) to different types of prostate cancer. While GWAS commonly the cutoff of 5% for minor allele frequency, recent publications suggest the potential contributions of less variants to the risk of different diseases have been discussed [42, 43]. It is believed that most of the heterozygosity in the human genome comes from in variants with a minor allele frequency. The fact that majority of detected splice-disrupt genomic variants in this study were minor variants supports this statement (96.01% in PC, 95.83 in FPC, 94.64 in CRP, and 97.14% in MCRP, Supplementary 1).

Apoptosis and angiogenesis are the main cancer-associated processes, where alternative splicing plays a crucial role in their regulation [44]. Functional annotation of high-risk splice-disrupt variants showed that they are mainly involved in endocrine resistance, regulation of mitotic cell cycle, negative and response to lipid that are involved in apoptosis and angiogenesis. We observed the enrichment of negative regulation of production of miRNAs involved in gene silencing, where *ESR1* are *NCOR1* are involved in this function.

Metastasis is the cause of more than 90% of cancer-related deaths and is the most complex function of cancer cells [7]. Metastasis requires phenotypic plasticity that is centred around epithelial–mesenchymal transition (EMT) [45]. Alternative splicing of several genes is shown to be linked to EMT, such as RBFox2 and ESRP [46, 47]. Shapiro et al. identified the first alternative splicing signature for EMT and showed that the key drivers of EMT, such as cytoskeleton remodelling, regulation of cell–cell junction formation, and regulation of cell migration, all experience alternative splicing events [48]. Recently, it has been reported that overexpression of *PTPRC* is involved in cell adhesion, facilitating the tumour proliferation and lymph node metastasis in cervical cancer patients [49]. In another study, the inhibition of *PTPRC* reduced the rates of tumour growth and metastasis in vivo [50]. The role of *PTPRC* has also been noticed in colon cancer metastasis [51]. It has been suggested that *PTPRC*, as an adhesion molecule, is involved in the spread of the tumour and immortalisation of the tumour cells during malignancy [52]. In gastric cancer, it has been shown that *CTSF* is involved in the growth and apoptosis where *CTSF* knockdown promotes proliferation by inhibiting apoptosis [53]. It has been suggested in *CTSF* gene may function as a tumour suppressor with high potential therapeutic value [53]. To best of our knowledge, this is the first report of splice-disrupt variants on *PTPRC* and *CTSF* genes and their involvement in prostate cancer. We found that splice-disrupt variants on *CTSF* and *PTPRC* is specific to MCRPC and may contribute to prostate cancer progression.

This is the first study that profiles the splice-disrupt genomic variants and their target genes in prostate cancer. Noticeably, we found an association between specific splice-disrupt variants with advanced prostate cancer. The major limitation of this study was the remarkable difference between number of splice-disrupt genomic in different types of prostate cancer. The variants in FPC, CRPC, and MCRPC are more detrimental and harder to detect compared to early stage of PC. Alternative splicing contributes to a range of phenotypic traits of tumours as they progress and undergo metastasis and is a potential target for gene therapy [7, 11]. Unravelling alternative splicing opens a new avenue towards the establishment of new diagnostic and prognostic markers for prostate cancer progression and metastasis [48], as well as the development of a new generation of anticancer therapeutics: Treatments that inhibit specific splice variants, rather than targeting genes.

## Electronic Supplementary Material

Below is the link to the electronic supplementary material.

Supplementary file 1 (DOCX 98 KB) List of splice-disrupt variantsin different types of prostate cancer. Prostate cancer (PC), castration-resistant prostate cancer (CRPC), and metastatic castration-resistant prostate cancer (MCRPC)

Supplementary file 2 (DOCX 25 KB) High-risk splice-disrupt variants in prostate cancer, based on PolyPhen, SIFT, GERP++ scores and reported clinical significance in dbSNP

Supplementary file 3 (DOCX 23 KB) High-risk splice-disrupt variants in familial prostate cancer (FPC) based on PolyPhen, SIFT, and GERP++ scores as well as reported clinical significance

Supplementary file 4 (DOCX 23 KB) High-risk splice-disrupt variants in castration-resistant prostate cancer (CRPC) based on PolyPhen, SIFT, and GERP++ scores as well as reported clinical significance

Supplementary file 5 (DOCX 23 KB) High-risk splice-disrupt variants in metastatic castration-resistant prostate cancer (MCRPC) based on PolyPhen, SIFT, and GERP++ scores as well as reported clinical significance

Supplementary file 6 (DOCX 24 KB) Functional annotation of high-risk splice-disrupt variants by enrichment analysis

Supplementary file 7 (XLSX 53 KB) Mined references underpinning literature-mining network of splice-disrupt variants, their annotated genes, and interactions with different types of prostate cancer. The network of relationships/interaction is visualised in Figure 6)

Supplementary file 8 (XLSX 11 KB)

Supplementary file 9 (DOCX 13 KB)
