# Supplementary material for: Splice-disrupt genomic variants in prostate cancer
Source: Mol Biol Rep. 2022 Mar 14;49(6):4237–46. doi: 10.1007/s11033-022-07257-9 (PMC9262760; doi:10.1007/s11033-022-07257-9)
Supplement: Supplementary file 2 — (DOCX 25 KB) High-risk splice-disrupt variants in prostate cancer, based on PolyPhen, SIFT, GERP++ scores and reported clinical significance in dbSNP [file 11033_2022_7257_MOESM2_ESM.docx]

**Supplementary 2**. High-risk splice-disrupt variants in prostate cancer, based on PolyPhen, SIFT, GERP++ scores and reported clinical significance

| **rsId** | **Chr.** | **Location** | **ref** | **Alt.** | **Gene** | **Gene region** | **GERP++ Score** | **SIFTScore** | **PolyPhen2 Score** | **Allele Frequency** | **Clin. Significance** |
| --- | --- | --- | --- | --- | --- | --- | --- | --- | --- | --- | --- |
| rs201633542 | 1 | 156811987 | T | C | *INSRR* | CDS, Intron | 4.68 | 0 | 1 | 0.0002 |  |
| rs200170667 | 6 | 30673059 | C | G | *MDC1* | CDS | 1.28 | 0 | 0.213 | 0.0002 |  |
| rs376935572 | 16 | 79245645 | G | A | *WWOX* | CDS | -5.5 | 0 | 0.006 | 0.0008 |  |
| rs200293570 | 4 | 1330780 | A | T | *MAEA* | CDS, 5UTR | -6.26 | 0 | 0 | 0.0002 |  |
| rs146032538 | 6 | 35610590 | A | G | *FKBP5* | CDS | 2.49 | 0 |  | 0.0184 |  |
| rs550790268 | 8 | 22960696 | T | A | *TNFRSF10C* | 5UTR, Intron | 1.62 | 0 |  | 0.0002 |  |
| rs557303188 | 10 | 61011334 | C | G | *FAM13C* | Intron | 5.74 | 0.001 | 1 | 0.0002 |  |
| rs188957694 | 6 | 152265353 | G | A | *ESR1* | CDS | 5.51 | 0.001 | 1 | 0.0002 |  |
| rs117054298 | 7 | 45932563 | A | T | *IGFBP1* | CDS | 4.57 | 0.001 | 0.999 | 0.001 |  |
| rs143384572 | 14 | 93185114 | C | T | *LGMN* | CDS | 5.52 | 0.001 | 0.998 | 0.0006 |  |
| rs397515354 | 2 | 219677473 | G | A | *CYP27A1* | Intron | 6.15 |  |  | 0.0002 | pathogenic |
| rs397515354 | 2 | 219677473 | G | C | *CYP27A1* | Intron | 6.15 |  |  | 0.0002 | pathogenic |
| rs75096551 | 7 | 117246808 | G | A | *CFTR* | Intron | 5.4 |  |  | 0.0002 | pathogenic |
| rs3918290 | 1 | 97915614 | C | T | *DPYD* | Intron | 5.31 |  |  | 0.003 | pathogenic |
| rs80358027 | 17 | 41234420 | C | A | *BRCA1* | Intron | 5.26 |  |  | 0.0002 | pathogenic |
| rs80358027 | 17 | 41234420 | C | G | *BRCA1* | Intron | 5.26 |  |  | 0.0002 | pathogenic |
| rs80358027 | 17 | 41234420 | C | T | *BRCA1* | Intron | 5.26 |  |  | 0.0002 | pathogenic |
| rs76713772 | 7 | 117227792 | G | A | *CFTR* | Intron | 5.06 |  |  | 0.0004 | pathogenic |
| rs267607789 | 3 | 37056036 | G | A | *MLH1* | Intron | 4.94 |  |  | 0.0002 | pathogenic |
| rs267607789 | 3 | 37056036 | G | C | *MLH1* | Intron | 4.94 |  |  | 0.0002 | pathogenic |
| rs148954387 | 5 | 147207583 | A | G | *SPINK1* | Intron | 4.9 |  |  | 0.0008 | pathogenic |
| rs191107774 | 11 | 68579904 | C | T | *CPT1A* | Intron | 4.59 |  |  | 0.0002 | pathogenic |
| rs3892097 | 22 | 42524947 | C | T | *CYP2D6* | Intron | 3.37 |  |  | 0.0931 | pathogenic |
