# Supplementary material for: Splice-disrupt genomic variants in prostate cancer
Source: Mol Biol Rep. 2022 Mar 14;49(6):4237–46. doi: 10.1007/s11033-022-07257-9 (PMC9262760; doi:10.1007/s11033-022-07257-9)
Supplement: Supplementary file 3 — (DOCX 23 KB) High-risk splice-disrupt variants in familial prostate cancer (FPC) based on PolyPhen, SIFT, and GERP++ scores as well as reported clinical significance [file 11033_2022_7257_MOESM3_ESM.docx]

**Supplementary 3**. High-risk splice-disrupt variants in familial prostate cancer (FPC) based on PolyPhen, SIFT, and GERP++ scores as well as reported clinical significance

| **rsId** | **Chromosome** | **Location** | **ref** | **Alt.** | **Gene** | **Gene region** | **GERP++ Score** | **SIFTScore** | **PolyPhen2 Score** | **Allele Frequency** | **Clin. Significance** |
| --- | --- | --- | --- | --- | --- | --- | --- | --- | --- | --- | --- |
| rs80358027 | 17 | 41234420 | C | A | *BRCA1* | Intron | 5.26 |  |  | 0.0002 | pathogenic |
| rs80358027 | 17 | 41234420 | C | G | *BRCA1* | Intron | 5.26 |  |  | 0.0002 | pathogenic |
| rs80358027 | 17 | 41234420 | C | T | *BRCA1* | Intron | 5.26 |  |  | 0.0002 | pathogenic |
| rs267607789 | 3 | 37056036 | G | A | *MLH1* | Intron | 4.94 |  |  | 0.0002 | pathogenic |
| rs267607789 | 3 | 37056036 | G | C | *MLH1* | Intron | 4.94 |  |  | 0.0002 | pathogenic |
| rs150131889 | 8 | 16035394 | C | A | *MSR1* | Intron | 5.36 |  |  | 0.0004 |  |
| rs33912200 | 8 | 16035502 | C | T | *MSR1* | Intron | 5.36 |  |  | 0.0012 |  |
| rs573868194 | 15 | 75013395 | C | A | *CYP1A1* | Intron | 5.17 |  |  | 0.0002 |  |
| rs545982789 | 22 | 29107895 | A | G | *CHEK2* | Intron | 5.07 |  |  | 0.0002 |  |
| rs574164122 | 17 | 12908305 | C | T | *ELAC2* | Intron | 4.9 |  |  | 0.0002 |  |
