# Supplementary material for: Splice-disrupt genomic variants in prostate cancer
Source: Mol Biol Rep. 2022 Mar 14;49(6):4237–46. doi: 10.1007/s11033-022-07257-9 (PMC9262760; doi:10.1007/s11033-022-07257-9)
Supplement: Supplementary file 4 — (DOCX 23 KB) High-risk splice-disrupt variants in castration-resistant prostate cancer (CRPC) based on PolyPhen, SIFT, and GERP++ scores as well as reported clinical significance [file 11033_2022_7257_MOESM4_ESM.docx]

**Supplementary 4**. High-risk splice-disrupt variants in castration-resistant prostate cancer (CRPC) based on PolyPhen, SIFT, and GERP++ scores as well as reported clinical significance

| **rsId** | **Chromosome** | **Location** | **ref** | **Alt.** | **Gene** | **Gene region** | **GERP++ Score** | **SIFTScore** | **PolyPhen2 Score** | **Allele Frequency** | **Clin. Significance** |
| --- | --- | --- | --- | --- | --- | --- | --- | --- | --- | --- | --- |
| rs201633542 | 1 | 156811987 | T | C | INSRR | CDS, Intron | 4.68 | 0 | 1 | 0.0002 |  |
| rs200293570 | 4 | 1330780 | A | T | MAEA | CDS, 5UTR | -6.26 | 0 | 0 | 0.0002 |  |
| rs188957694 | 6 | 152265353 | G | A | ESR1 | CDS | 5.51 | 0.001 | 1 | 0.0002 |  |
| rs142712646 | 6 | 152265352 | C | T | ESR1 | CDS | 5.51 | 0.008 | 1 | 0.0008 |  |
| rs564839203 | 10 | 123971010 | A | C | TACC2 | CDS | 4.74 | 0.031 | 0.999 | 0.0002 |  |
| rs564059250 | 13 | 48878068 | G | A | RB1 | CDS | 3.95 | 0.228 | 0.994 | 0.0002 |  |
| rs34672691 | 1 | 159683791 | A | G | CRP | CDS | 2.81 | 0.408 | 0.049 | 0.0012 |  |
| rs80358027 | 17 | 41234420 | C | A | BRCA1 | Intron | 5.26 |  |  | 0.0002 | pathogenic |
| rs80358027 | 17 | 41234420 | C | G | BRCA1 | Intron | 5.26 |  |  | 0.0002 | pathogenic |
| rs80358027 | 17 | 41234420 | C | T | BRCA1 | Intron | 5.26 |  |  | 0.0002 | pathogenic |
