# Supplementary material for: Splice-disrupt genomic variants in prostate cancer
Source: Mol Biol Rep. 2022 Mar 14;49(6):4237–46. doi: 10.1007/s11033-022-07257-9 (PMC9262760; doi:10.1007/s11033-022-07257-9)
Supplement: Supplementary file 5 — (DOCX 23 KB) High-risk splice-disrupt variants in metastatic castration-resistant prostate cancer (MCRPC) based on PolyPhen, SIFT, and GERP++ scores as well as reported clinical significance [file 11033_2022_7257_MOESM5_ESM.docx]

**Supplementary 5**. High-risk splice-disrupt variants in metastatic castration-resistant prostate cancer (MCRPC) based on PolyPhen, SIFT, and GERP++ scores as well as reported clinical significance

| **rsId** | **Chromosome** | **Location** | **ref** | **Alt.** | **Gene** | **Gene region** | **GERP++ Score** | **SIFTScore** | **PolyPhen2 Score** | **Allele Frequency** | **Clin. Significance** |
| --- | --- | --- | --- | --- | --- | --- | --- | --- | --- | --- | --- |
| rs201362929 | 12 | 124846841 | C | G | NCOR2 | CDS | 4.91 | 0.001 | 0.99 | 0.0002 |  |
| rs202028951 | 12 | 124857015 | G | A | NCOR2 | CDS | 4.63 | 0.008 | 0.022 | 0.0002 |  |
| rs527250408 | 1 | 198663271 | T | A | PTPRC | CDS, Intron | 0.225 | 0 |  | 0.0002 |  |
| rs34672691 | 1 | 159683791 | A | G | CRP | CDS | 2.81 | 0.408 | 0.049 | 0.0012 |  |
| rs78370249 | 11 | 115110993 | C | T | CADM1 | Intron | 5.97 |  |  | 0.0002 |  |
| rs188703146 | 7 | 55221703 | G | C | EGFR | Intron | 5.81 |  |  | 0.0002 |  |
| rs200215334 | 17 | 15983379 | C | G | NCOR1 | Intron | 5.79 |  |  | 0.0002 |  |
| rs80358027 | 17 | 41234420 | C | A | BRCA1 | Intron | 5.26 |  |  | 0.0002 | pathogenic |
| rs80358027 | 17 | 41234420 | C | G | BRCA1 | Intron | 5.26 |  |  | 0.0002 | pathogenic |
| rs80358027 | 17 | 41234420 | C | T | BRCA1 | Intron | 5.26 |  |  | 0.0002 | pathogenic |
