# Supplementary material for: Splice-disrupt genomic variants in prostate cancer
Source: Mol Biol Rep. 2022 Mar 14;49(6):4237–46. doi: 10.1007/s11033-022-07257-9 (PMC9262760; doi:10.1007/s11033-022-07257-9)
Supplement: Supplementary file 6 — (DOCX 24 KB) Functional annotation of high-risk splice-disrupt variants by enrichment analysis [file 11033_2022_7257_MOESM6_ESM.docx]

**Supplementary 6**. Functional annotation of high-risk splice-disrupt variants by enrichment analysis

| **Function** | **term description** | **false discovery rate** | **matching proteins Iwith slice-disrupt variant** |
| --- | --- | --- | --- |
| Biological Process | steroid metabolic process | 0.00018 | CFTR,CYP1A1,CYP27A1,CYP2D6,ESR1,LGMN,WWOX |
| Biological Process | lipid metabolic process | 0.0028 | BRCA1,CFTR,CPT1A,CYP1A1,CYP27A1,CYP2D6,EGFR,ESR1,LGMN,WWOX |
| Biological Process | regulation of mitotic cell cycle | 0.0028 | BRCA1,CHEK2,CYP1A1,EGFR,LGMN,MAEA,MDC1,RB1 |
| Biological Process | negative regulation of metabolic process | 0.0028 | BRCA1,CHEK2,CYP2D6,EGFR,ESR1,LGMN,MAEA,MLH1,MSR1,NCOR1,PTPRC,RB1,SPINK1,TNFRSF10C,WWOX |
| Biological Process | negative regulation of signal transduction | 0.0028 | BRCA1,EGFR,ESR1,IGFBP1,LGMN,NCOR1,PTPRC,RB1,SPINK1,WWOX |
| Biological Process | response to lipid | 0.0028 | BRCA1,CFTR,CPT1A,CYP1A1,EGFR,ESR1,RB1,TNFRSF10C |
| Biological Process | positive regulation of cell cycle | 0.0028 | BRCA1,CHEK2,CYP1A1,EGFR,LGMN,RB1 |
| Biological Process | negative regulation of biological process | 0.0028 | BRCA1,CHEK2,CRP,CYP2D6,EGFR,ESR1,IGFBP1,LGMN,MAEA,MDC1,MLH1,MSR1,NCOR1,NTRK1,PTPRC,RB1,SPINK1,TNFRSF10C,WWOX |
| Biological Process | negative regulation of cellular process | 0.0028 | BRCA1,CHEK2,CRP,CYP2D6,EGFR,ESR1,IGFBP1,LGMN,MAEA,MDC1,MLH1,NCOR1,NTRK1,PTPRC,RB1,SPINK1,TNFRSF10C,WWOX |
| Biological Process | negative regulation of response to stimulus | 0.0028 | BRCA1,CHEK2,EGFR,ESR1,IGFBP1,LGMN,NCOR1,PTPRC,RB1,SPINK1,WWOX |
|  |  |  |  |
| Molecular Function | nuclear hormone receptor binding | 0.00096 | BRCA1,ESR1,NCOR1,RB1,TACC2 |
| Molecular Function | protein binding | 0.0011 | BRCA1,CADM1,CFTR,CHEK2,CPT1A,CRP,CYP1A1,DPYD,EGFR,ESR1,FKBP5,IGFBP1,INSRR,MAEA,MDC1,NCOR1,NTRK1,PTPRC,RB1,TACC2,TNFRSF10C,WWOX |
| Molecular Function | steroid hydroxylase activity | 0.0014 | CYP1A1,CYP27A1,CYP2D6 |
| Molecular Function | nuclear receptor binding | 0.0015 | BRCA1,ESR1,NCOR1,RB1 |
| Molecular Function | signaling receptor binding | 0.0022 | BRCA1,CADM1,CRP,EGFR,ESR1,IGFBP1,NCOR1,NTRK1,RB1,TACC2 |
| Molecular Function | transmembrane receptor protein tyrosine kinase activity | 0.0044 | EGFR,INSRR,NTRK1 |
| Molecular Function | identical protein binding | 0.0053 | CADM1,CHEK2,CPT1A,CRP,DPYD,EGFR,ESR1,NTRK1,PTPRC,RB1 |
| Molecular Function | enzyme binding | 0.0065 | BRCA1,CFTR,CHEK2,CYP1A1,EGFR,ESR1,NCOR1,NTRK1,PTPRC,RB1,WWOX |
| Molecular Function | low-density lipoprotein particle binding | 0.0065 | CRP,MSR1 |
| Molecular Function | catalytic activity | 0.0066 | BRCA1,CFTR,CHEK2,CPT1A,CYP1A1,CYP27A1,CYP2D6,DPYD,EGFR,ELAC2,ESR1,FKBP5,INSRR,LGMN,MLH1,NTRK1,PTPRC,WWOX |
| Molecular Function | steroid hormone receptor binding | 0.0072 | BRCA1,ESR1,RB1 |
| Molecular Function | kinase binding | 0.008 | CHEK2,EGFR,ESR1,NTRK1,PTPRC,RB1 |
|  |  |  |  |
| KEGG pathway | Endocrine resistance | 4.31E-05 | CYP2D6,EGFR,ESR1,NCOR1,RB1 |
| KEGG pathway | Breast cancer | 0.0042 | BRCA1,EGFR,ESR1,RB1 |
| KEGG pathway | Prostate cancer | 0.0169 | EGFR,INSRR,RB1 |
| KEGG pathway | Estrogen signaling pathway | 0.0284 | EGFR,ESR1,FKBP5 |
| KEGG pathway | Pathways in cancer | 0.0284 | EGFR,ESR1,MLH1,NTRK1,RB1 |
| KEGG pathway | Gastric cancer | 0.0284 | EGFR,MLH1,RB1 |
| KEGG pathway | Bladder cancer | 0.0301 | EGFR,RB1 |
| KEGG pathway | Fanconi anemia pathway | 0.0398 | BRCA1,MLH1 |
| KEGG pathway | Endometrial cancer | 0.0451 | EGFR,MLH1 |
