# Supplementary material for: Splice-disrupt genomic variants in prostate cancer
Source: Mol Biol Rep. 2022 Mar 14;49(6):4237–46. doi: 10.1007/s11033-022-07257-9 (PMC9262760; doi:10.1007/s11033-022-07257-9)
Supplement: Supplementary file 9 — (DOCX 13 KB) [file 11033_2022_7257_MOESM9_ESM.docx]

**Electronic Supplementary Material**

**Supplementary 1.** List of splice-disrupt variants in different types of prostate cancer. Prostate cancer (PC), castration-resistant prostate cancer (CRPC), and metastatic castration-resistant prostate cancer (MCRPC)

**Supplementary 2**. High-risk splice-disrupt variants in prostate cancer, based on PolyPhen, SIFT, GERP++ scores and reported clinical significance in dbSNP.

**Supplementary 3**. High-risk splice-disrupt variants in familial prostate cancer (FPC) based on PolyPhen, SIFT, and GERP++ scores as well as reported clinical significance

**Supplementary 4**. High-risk splice-disrupt variants in castration-resistant prostate cancer (CRPC) based on PolyPhen, SIFT, and GERP++ scores as well as reported clinical significance

**Supplementary 5**. High-risk splice-disrupt variants in metastatic castration-resistant prostate cancer (MCRPC) based on PolyPhen, SIFT, and GERP++ scores as well as reported clinical significance

**Supplementary 6**. Functional annotation of high-risk splice-disrupt variants by enrichment analysis

**Supplementary 7.** Mined references underpinning literature-mining network of splice-disrupt variants, their annotated genes, and interactions with different types of prostate cancer. The network of relationships/interaction is visualised in Figure 6).

**Supplementary 8.** Mined references underpinning literature-mining network of rs1800716 splice-disrupt. The network of relationships/interaction is visualised in Figure 7.
